# A Neural Population Model Incorporating Dopaminergic Neurotransmission during Complex Voluntary Behaviors

**DOI:** 10.1371/journal.pcbi.1003924

**Published:** 2014-11-13

**Authors:** Stefan Fürtinger, Joel C. Zinn, Kristina Simonyan

**Affiliations:** 1 Department of Neurology, Icahn School of Medicine at Mount Sinai, New York, New York, United States of America; 2 Department of Otolaryngology, Icahn School of Medicine at Mount Sinai, New York, New York, United States of America; Universitat Pompeu Fabra, Spain

## Abstract

Assessing brain activity during complex voluntary motor behaviors that require the recruitment of multiple neural sites is a field of active research. Our current knowledge is primarily based on human brain imaging studies that have clear limitations in terms of temporal and spatial resolution. We developed a physiologically informed non-linear multi-compartment stochastic neural model to simulate functional brain activity coupled with neurotransmitter release during complex voluntary behavior, such as speech production. Due to its state-dependent modulation of neural firing, dopaminergic neurotransmission plays a key role in the organization of functional brain circuits controlling speech and language and thus has been incorporated in our neural population model. A rigorous mathematical proof establishing existence and uniqueness of solutions to the proposed model as well as a computationally efficient strategy to numerically approximate these solutions are presented. Simulated brain activity during the resting state and sentence production was analyzed using functional network connectivity, and graph theoretical techniques were employed to highlight differences between the two conditions. We demonstrate that our model successfully reproduces characteristic changes seen in empirical data between the resting state and speech production, and dopaminergic neurotransmission evokes pronounced changes in modeled functional connectivity by acting on the underlying biological stochastic neural model. Specifically, model and data networks in both speech and rest conditions share task-specific network features: both the simulated and empirical functional connectivity networks show an increase in nodal influence and segregation in speech over the resting state. These commonalities confirm that dopamine is a key neuromodulator of the functional connectome of speech control. Based on reproducible characteristic aspects of empirical data, we suggest a number of extensions of the proposed methodology building upon the current model.

## Author Summary

Our knowledge of brain activity and network organization during complex motor behaviors in humans relies mainly on neuroimaging studies. However, the majority of available brain imaging methods are not feasible for quantifying the neural processes that occur on very short time-scales at the microscopic level. To address this shortcoming of functional MRI, we designed a mathematical model, which simulates brain activity using local ensembles of neurons and physiologically meaningful variables, such as cellular membrane potentials and ion channel relaxation times. We further incorporated dopaminergic function into our model as a neuromodulator of the dynamic organization of brain networks. We applied our model to examine brain networks controlling human speech and language production. We present a rigorous mathematical proof, which establishes the theoretical validity and solvability of the presented model, and we discuss the influence of dopaminergic transmission on simulated brain activity. We show that our model successfully reproduces characteristic changes seen in empirical data between the resting state and speech production. Our results indicate that the proposed mathematical model may be used as a platform for future studies to investigate the specific impact of certain pathologies within the dopaminergic pathways and their effect on global network dynamics.

## Introduction

Computational neuroscience takes a grounds-up approach to understand complex neural phenomena by investigating the underlying activity of neurons themselves. Starting from the basic neural model of Hodgkin and Huxley [1], which described the electric activity of a neuron in terms of the cell's constituent ionic currents, computational neuroscientists began to study temporal input-output relations of neural units. One of the most famous models created during this era was the “point unit” by McCulloch and Pitts [2]. Among the first modelers who incorporated not only temporal but also spatial aspects of neural processing was Rall, who used compartment models to show the strong impact of dendritic arborization on neural processing of synaptic inputs [3]. His work laid the ground for the first neural network modeling based on rather complex single neuron models. Next, the so-called Wilson-Cowan units [4] allowed for simulation of rather realistically macroscopic responses of entire brain regions on the scales corresponding to measurements obtained by non-invasive *in vivo* human imaging techniques. Indeed, neural simulations did fit well with data from various human imaging modalities, such as magnetoencephalography (MEG) [5], positron emission tomography (PET) [6], and functional magnetic resonance imaging (fMRI) [7], [8]. Many neural states have been modeled, resulting in a rich literature on resting-state brain activity as well as behavior-specific activities, such as visual [6], memory [9], sensorimotor [10] and auditory [11] processing. However, while it is generally accepted that differences may be seen between the resting state and task conditions as well as between healthy and patient data and models, the fundamental question in modeling and data analysis methodology, the significance of differences between modeled conditions, remains unclear [12].

Given that functional activity may be affected by neurotransmitters (see [13] for a review), recent modeling efforts have been undertaken to integrate neuromodulators, such as dopamine, into task simulations. Dopaminergic neurotransmission has been implicated in cognition, learning, motor control, and, more generally, sensorimotor integration [14], [15], [16]. Chadderdon and Sporns proposed a large-scale computational model of prefrontal cortex to show the effects of dopamine release on the onset and performance of working memory tasks, which could be confirmed by behavioral, single-cell and neuroimaging data [17]. Determining how dopamine may regulate the functional connectivity observed during a behavioral task is a critical next step in addressing the ambiguity of task-specific functional connectivity.

To that end, we present a biologically-informed, large-scale model, which is based on neurobiological considerations, to simulate neuronal function and connectivity modulated by dopamine release in the human brain. The present paper applies this model to investigate speech production, one of the most complex human behaviors, which can be studied in a neuroimaging setting. Speech production is known to integrate several neural networks, ranging from auditory processing to motor control of articulatory movements [18], [19]. Our recent study has demonstrated that dopaminergic modulation may play a role in left-hemispheric lateralization of functional brain activity and connectivity during speech production in the absence of lateralized structural networks [20]. Here, we propose an extension of a non-linear model presented by Breakspear et al. [21] to allow for the simulation of brain activity due to dopaminergic modulation. We introduce the original model, which is a system of stochastic differential equations (SDEs), and rewrite it in terms of a multi-dimensional time-continuous stochastic process. Coupling between the brain regions with respect to regional neural firing rates is incorporated within the framework of Ito processes [[22], Chap. 7]. A dopamine release model is developed and integrated into the model linking the basal ganglia and the laryngeal motor cortex based on previous studies [23]. We further present a mathematical proof establishing the existence and uniqueness of solutions to the extended model. Exploiting specific structural properties of the model, a computationally efficient scheme for numerical approximation of solutions is also presented. We show simulations of resting-state and dopamine-modulated BOLD signals and analyze the associated functional connectivity networks as related to corresponding real fMRI data obtained from healthy volunteers during the resting state and speech production. Finally, we discuss merits and limitations of the proposed model.

## Materials and Methods

### Ethics Statement

All participants provided written informed consent before participation in the study, which was approved by the Institutional Review Boards of the Icahn School of Medicine at Mount Sinai and National Institute of Neurological Disorders and Stroke, National Institutes of Health.

### Modeling Objective

Our goal was to simulate a large-scale neural population using 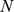 coupled small scale local models, each replicating neural activation in a specific brain region 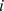 (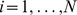), while incorporating neuromodulator release in a region-specific manner. Every regional subsystem consisted of interconnected excitatory and inhibitory neurons, which were assumed to be representatives of the local neural ensemble within a region. Thus, all quantities were understood as mean values across the considered region. The dynamics of regional state variables were governed by voltage-gated ion channels, functional synaptic couplings and neurotransmitter release. Thus, the temporal evolution of the entire population was determined solely by the interaction of its regional subsystems. In contrast to other approaches, the model discussed here was not based on coupled oscillator systems like the widely-used Kuramoto model (compare, e.g., [24] or [25]), but was based on neurobiological considerations. Below, we first detail the theoretical aspects of the model, including the Wilson-Cowan and dopamine dynamics, then describe the integration of the model with data.

### The Breakspear Neural Model

Following Breakspear et al. [21], we denote the average membrane potential of neurons in region 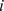 by 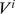, which we assume to be governed by voltage-gated potassium (K), sodium (Na) and calcium (Ca) ion channels together with the passive conductance of leaky (L) ions. Thus, for 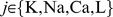 let 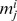 denote the fraction of open *j*-ion channels and let 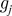 be the ion population's maximum conductance for 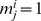 (i.e., when all *j*-ion channels are open). The basic model describing current flows across neural membranes in region 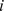 is a balance equation of the form (assuming unit neural capacitance) 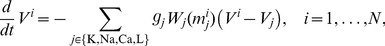
(1)where 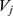 denote the respective Nernst potentials and 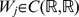 are neural activation functions. To adequately reflect relaxation times of potassium channels, 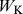 is characterized by an exponential decay 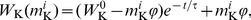
(2)with 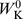 being the value of 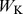 at the initial time 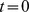, 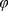 denoting a temperature scaling factor and 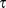 being the relaxation time. For brevity, we introduce the shorthand notation 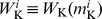. The other neural activation functions are defined as 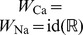 and 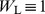.

Assuming that the ion channel specific opening-thresholds are normally distributed with mean 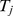 and variance 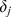 across the considered neural population, the fraction of open channels in region 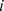 may be computed as 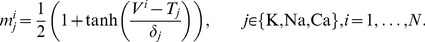
(3)


Note that the basic model (1) consists exclusively of uncoupled equations, i.e., the membrane potential of neurons in region 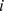 is entirely independent of neural firing in neighboring regions. Coupling is introduced by considering firing rates of excitatory and inhibitory neurons across the whole population. Thus let 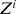 be the mean membrane potential of inhibitory interneurons in region 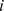, and define average excitatory and inhibitory firing rates as follows 
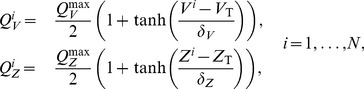
(4)where 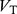 and 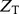 denote the mean values and 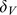 and 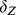 are the variances of membrane threshold potentials of excitatory and inhibitory neurons, respectively (assuming a normal distribution of thresholds across the neural population). Regional membrane potentials are altered by excitatory and inhibitory cell firing via synaptic feedback loops. Thus, functional synaptic factors 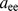, 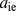 and 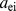 are introduced to scale excitatory-to-excitatory, inhibitory-to-excitatory and excitatory-to-inhibitory couplings, respectively. Furthermore, to reflect firing rate dependent glutamate neurotransmitter release (opening additional calcium ion channels) a supplemental scaling parameter 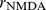 (the ratio of NMDA to AMPA receptors) is used. Non-specific input to excitatory and inhibitory neurons is modeled using random noise, which gives rise to a system of coupled stochastic differential equations (SDEs). Thus let 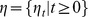 denote a scalar Wiener process [[26], Sec. 1.6] and let 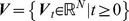 and 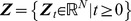 be 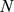-dimensional Ito processes. To avoid notational overhead we understand the auxiliary quantities (3) and (4) to be obviously adapted to 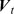 (replace 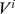 by the components 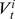 of 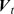 in the respective definitions). In the following we establish a vectorial representation of the basic model equations given in [21]. Thus, for a vector 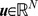 let 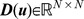 denote a diagonal matrix with the components of 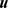 on its main diagonal. Further, we introduce the 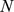-dimensional vectors 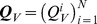, 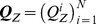, 
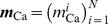
, 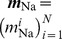 and 
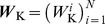
. With 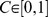 denoting a (global) coupling parameter and 
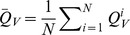
, we define a function 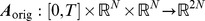 (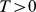) with components 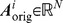 given by 
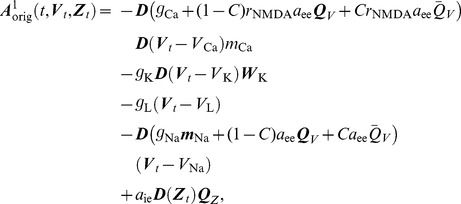
(5)and 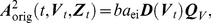
(6)such that 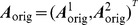. Similarly with 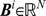 we introduce a vector 
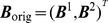
 defined by 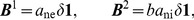
(7)where 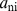 and 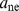 denote synaptic factors corresponding to non-specific excitatory/inhibitory input, 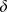 is a noise scaling parameter and 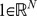 is a vector of ones. Setting 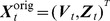 we thus obtain the SDE 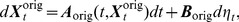
(8)which is the Ito version of the original multi-compartment neural dynamics model presented by Breakspear et al. [21].

### Model Extension 1: Inter-Regional Connectivity

The original model (8) uses a scalar parameter 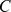 to parameterize excitatory coupling between regions. This means, in the framework considered here, that inter-regional connectivity strengths constant throughout the entire brain. To relax this restrictive assumption, we assign each pair of regions 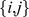 coupling parameters 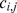 and 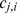 representing the connectivity strengths 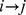 and 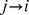, respectively. We collect the inter-regional coupling parameters in a 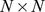 matrix 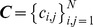 and incorporate it in the model (8) as follows. Instead of calculating excitatory-to-excitatory neural feedback by relying on a mean firing rate 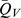, we scale neural firing using weight information from the coupling matrix 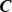. Thus, we assume that firing of brain areas connected to region 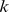 impact the membrane potential in region 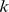 according to 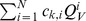. Hence, (5) is modified to be 
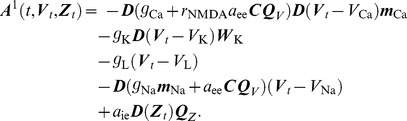
(9)


We set 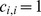 to reflect local excitatory input within a region. Note that we do not impose any restrictions on the directionality of regional couplings. Depending on the application considered, the above formulation allows the use of directed connections (i.e., a non-symmetric coupling matrix 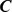) or undirected connections (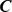 symmetric).

### Model Extension 2: A Dopamine Release Model

A second extension to the original model was incorporated to simulate the effects of speech-induced dopamine release, as shown previously in real data [20]. We were especially interested in the effect of dopaminergic neurotransmission on the primary motor cortex [27] and its direct influence on the activity of the laryngeal motor cortex (LMC), which is a final common cortical pathway of speech control [28], [29]. Keeping in mind the biologically-inspired channel model adopted in the present paper, elevated dopamine levels in the striatum (without a differential effect on either D1 or D2 type of dopamine receptors) were assumed to increase the probability that potassium, sodium and calcium channels of LMC neurons open, thus making these neurons more likely to fire. Hence, we simulated both D1- and D2-type modulatory effects on these channels [30].

We modeled the direct dopaminergic pathway from the substantia nigra, pars compacta (SNc) to the LMC [28]. Thus, we assumed that dopamine release was solely driven by neural activity in the SNc. Hence, let 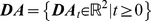 be a two-dimensional Ito process with components 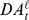 and 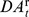 denoting the dopamine concentration in the left (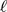) and right (r) LMC respectively. We assume 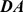 is governed by two simple mass balance equations 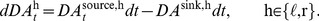
(10)


To reflect the positive feedback of neural firing in the SNc on dopamine release we define 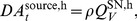
(11)where 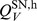 denotes the neural firing rate in the left/right substantia nigra as defined in (4), and 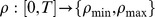 is a (time-defpendent) production rate. We assume that 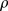 attains a maximum value 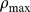 during speech production and is equal to a (positive) minimum value 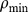 otherwise. The precise value of the uptake rate is taken to be a reasonable value from previous studies of extracellular dopamine levels [31]. Following [32], dopamine re-uptake was presumed to be governed by a Michaelis-Menten type kinetics equation 
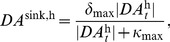
(12)where 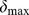 denotes the maximal uptake rate and 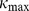 is the Michaelis-Menten constant. Thus, a closed form representation of the considered dopamine model is 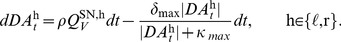
(13)


As mentioned above, dopamine was assumed to affect the firing of the LMC by altering neural ion channel permeability. Thus, the effect of dopamine on potassium channels can be seen as a dependence of the gain in 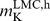 on dopamine concentration. Hence we modify the equation governing the fraction of open potassium channels in the LMC as follows 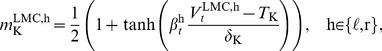
(14)where 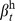 denotes a dopamine dependent gain. In the absence of dopamine we want a gain of unity, i.e., 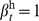. Conversely, we also like to impose an upper bound on the gain. To achieve this, consider the expression 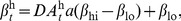
(15)where 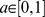 is an antagonism parameter controlling the overall impact of dopamine on the gain 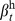. Obviously, if 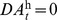 then 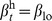, thus, by setting 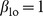, a unity gain for a dopamine concentration of zero is established. Since 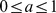 and assuming physiologically meaningful dopamine concentrations, i.e., 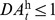, 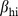 sets an upper bound for the gain.

Finally, we modeled the impact of dopamine on calcium and sodium channels in the LMC using the gain 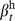. We expressed the dopamine dependence of the permeability of those channels via varying the LMC's excitatory-to-excitatory functional synaptic coupling by introducing 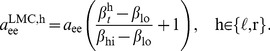
(16)


In the absence of dopamine we have 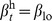 and thus 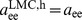. Rising dopamine levels increase 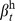 and, in turn, 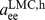 until 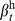 reaches its previously established upper bound 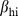, which gives 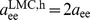. Thus, we have the estimate 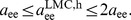
(17)


To establish a closed form representation of the full model, let 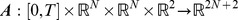 be defined by 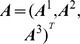, where 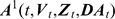 is given by the right hand side of (9) with LMC components 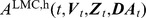 defined by 
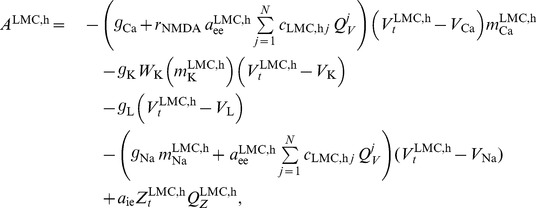
(18)for 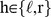. Let further 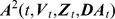 be given by the right hand side of (6) and define 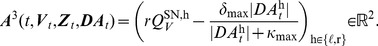
(19)


Similarly, let 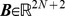 be given by 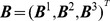 with 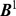 and 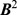 as defined in (7) and 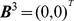. Then with 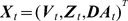 the full neural dynamics model can be written as 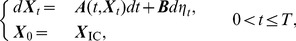
(20)where 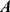 is called drift (or deterministic force) and 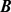 is the diffusion (or random force) of the model. In the following section we discuss an efficient strategy to numerically approximate solutions to the model (20). A rigorous mathematical proof establishing existence and uniqueness of those solutions is presented later.

### Numerical Approximation

We used time discrete approximation techniques to simulate sample paths of the SDE system (20). Extensive numerical experiments revealed pronounced non-linear dynamics of the model, which motivated the use of a higher order solution scheme. We encountered numerical instability of the widely used strong order 1.0 Milstein scheme [33]. Using a strong order 1.5 explicit Runge-Kutta (RK15) method, however, proved to be reliable. An explicit strong order 2.0 scheme yielded no notable improvements over the RK15 method but required a considerably higher computational effort. Thus, a RK15 scheme was specifically adapted to the model (20).

To establish a time discrete approximation of the solution to (20), we started by defining a discretization of the interval 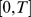. For 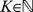, let 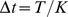 be a step-size and define discrete time points 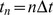 for 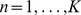. We introduce the Markov chain 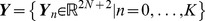 to approximate the stochastic process 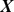 that satisfies (20). Thus we set 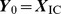 and 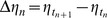. Note that (20) is a 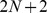-dimensional non-autonomous SDE with constant additive scalar noise. This latter property is exploited to construct a highly efficient recursive solution scheme that has a considerably reduced computational cost compared to a general purpose SDE solver.

The following considerations are based on the family of solution schemes presented in [[26], Sec. 11.2]. The vector form of an explicit order 1.5 strong scheme for a non-autonomous SDE with constant additive scalar noise is given by 
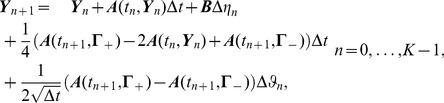
(21)where 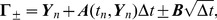
(22)and 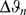 is a random variable representing the following double stochastic integral 
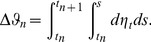
(23)


Rearranging terms, (21) can be simplified to 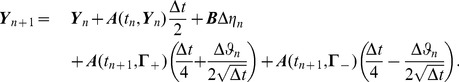
(24)


Note that 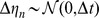 and 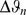 is also normally distributed satisfying 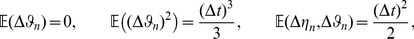
(25)as shown in [[26], Chap. 10]. These properties play a key role in practice since they allow us to generate the pair of correlated random variables 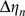 and 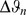 in an efficient and straight forward manner: let 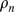 and 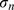 be independent 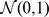 distributed random variables, then 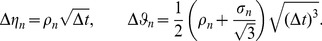
(26)


Thus, an approximate solution of (20) was recursively computed following scheme (24) with auxiliary quantities (22) and (26).

Note that (24) requires three evaluations of the drift term 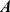 per step. In contrast, the Milstein method adapted to model (20) reduces to 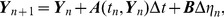
(27)and thus requires only one function evaluation per step. However, unlike RK15, (27) reduces to an explicit Euler scheme in the absence of noise (zero diffusion). Thus numerical instability of the Milstein scheme for a model like (20) exhibiting pronounced non-linear characteristics in the drift term was predictable. Note that it is possible to enforce convergence of (27) by substantially reducing the step-size 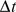. However, this in turn dramatically increases the total number of time-steps making the overall computational performance of the Milstein method significantly worse than that of RK15 (24). Hence RK15 was the solver of choice for all simulations presented below.

In order to produce measureable changes in extracellular dopamine levels, which reflect rapid phasic dopamine release during a behavioral task or a pharmacological challenge, the dopaminergic axons must be stimulated at frequencies of 10-20 Hz or greater [34]. Because phasic dopamine release may reach high concentrations for brief periods due to concerted burst firing of dopamine neurons [34], [35], [36], we tested our model at a neural firing rate>20 Hz with different time-step sizes. We found that a small step-size of 0.1ms had the highest numerical robustness and showed the optimal temporal resolution of neural firing in order for dopamine release/re-uptake to set in gradually, without jumps.

The simulations shown below have been run on a Mid 2010 Mac Pro (2×2.66 GHz 6-Core Intel Xeon, 24GB DDR3 ECC RAM) under OS X 10.9.1. All codes have been written in Python [37] making extensive use of the packages NumPy, SciPy [38] and Matplotlib [39]. Computationally expensive sections of the code have been converted to C extensions using Cython [40].

### Integration of Model and Data

#### Data acquisition

**Figure 1:**
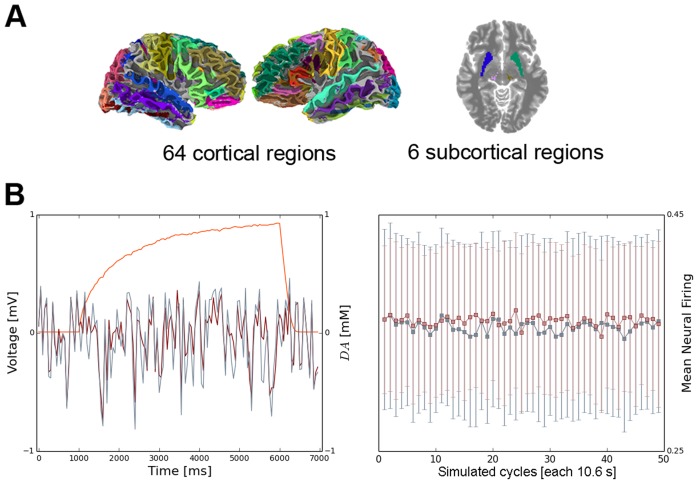
(A) Schematic overview of the whole-brain parcellations and (B) temporal evolution of LMC membrane potentials and firing rates. (A) The whole brain was parcellated into 70 regions of interest, including 64 cortical and 6 subcortical areas. (B) The left panel shows the time-course of excitatory membrane potentials 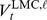 with (red) and without (blue) dopamine modulation overlayed with the corresponding time-evolution of 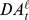 (orange) during one dopamine release cycle. The right panel illustrates the evolution of 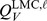 with (red) and without (blue) dopamine modulation for fifty simulated cycles (each 10.6 s). Boxes indicate mean firing rate values averaged across a cycle, errorbars show corresponding standard deviations.

The raw model output was converted to a blood oxygen level-dependent (BOLD) signal and compared to functional brain activity data in healthy volunteers. We used fMRI data of 20 right-handed monolingual English speaking subjects with no history of neurological, psychiatric, voice, or respiratory problems (13 females, 7 males, age 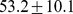 years [mean
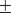
SD]) as reported earlier [20]. Right-handed volunteers were recruited in order to control for brain activity lateralization differences between right- and left-handed people. All scanning sessions were performed on a 3.0 Tesla GE scanner equipped with a quadrature birdcage radio frequency head coil (Milwaukee, WI). Data were acquired under two conditions: 1) a resting state, during which the subjects fixated on a cross, and 2) a task production, during which subjects were asked to produce meaningful, grammatically-correct, short sentences. Whole-brain resting-state (rs-fMRI) images were acquired using gradient-weighted echo planar imaging (EPI) (150 contiguous volumes with TR 2 s, TE 30 ms, FA 90 degrees, 33 sagittal slices, slice thickness 4 mm, matrix 64×64 mm, FOV 240 mm, in-plane resolution 3.75 mm, duration 5 min). To assure the resting condition, these images were acquired before the task-production fMRI within the same scanning session. Physiological recordings were carried out using a respiratory belt to measure respiration volume and a pulse oximeter to monitor heart rhythm and were sampled at 50 Hz with the recording onset triggered by the scanner's selection trigger pulse. For speech-production fMRI, whole brain images were acquired using gradient-weighted EPI pulse sequences (TE 30ms, TR 10.6 s (8.6 s task production, 2 s image acquisition), FA 90 degrees, FOV 240×240 mm, matrix 64×64 mm, in-plane resolution 3.75 mm, 33 sagittal slices, slice thickness 4.0 mm) with BOLD contrast and a sparse-sampling event-related design. The subjects were instructed to produce short meaningful grammatically correct English sentences (e.g., “We are always away”, “Tom is in the army”) after listening to an auditory sample. The auditory stimuli were delivered within a 3.6 s-period and the subjects reproduced the sentences within 5 s, followed by a 2-s image acquisition. A total of 36 trials per task (i.e., sentences, resting fixation) were acquired over the five scanning sessions with the tasks pseudorandomized between sessions and participants. All fMRI data was pre-processed using AFNI software package [41]. For rs-fMRI, the anatomy-based correlation corrections (ANATICOR) model [42] was applied to remove hardware-related noise; respiratory and cardiac signals synchronized with the EPI data were used to remove physiological noise based on the retrospective image correction (RETROICOR) model [43]. The resting-state residual time series were spatially smoothed by a 6-mm Gaussian kernel within the gray matter and normalized to the standard Talairach-Tournoux space. Task-production fMRI. For speech-production fMRI, the first two volumes were discarded, the EPI datasets were registered to the volume collected closest in time to the high-resolution anatomical scan using heptic polynomial interpolation, spatially smoothed with a 6-mm Gaussian filter, normalized to the percent signal change and the standard Talairach-Tournoux space. The task-related responses were analyzed using multiple linear regression with a single regressor for the task convolved with a canonical hemodynamic response function. Based on empirical studies [44], [45], the whole brain was parcellated into 70 regions of interest (ROIs), including 64 cortical and 6 subcortical areas (Fig. 1A).

#### Coupling Matrix

The coupling matrix 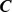 was based on anatomical connectivity estimated from fiber tractography using diffusion weighted data from nine out of twenty healthy subjects described above. A single-shot spin-echo EPI sequence with TE 80 ms, TR 8.9 s, FOV 240 mm, matrix 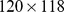 mm, 68 contiguous axial slices, slice thickness 2 mm was used to acquire whole-brain diffusion-weighted images. A total of 60 noncollinear directions with a b-factor of 1,000 s/mm^2^ were used to measure diffusion. One reference image was acquired with no diffusion gradients applied (b0 scan). Based on the same 70 ROIs, the DTI data were processed using the FATCAT Toolbox of AFNI software [46] following standard steps to construct an averaged 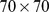 structural connectivity matrix. The matrix was normalized with respect to its largest row-sum and used as coupling matrix 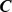 for all simulations presented below.

## Results

Below, we start by showing existence and uniqueness of solutions to the model (20) (Theorem 1). Once this fundamental result is established, we present simulations generated by the model and analyze it with respect to empirical fMRI data.

### Existence and Uniqueness of Solutions to the Neural Dynamics Model

The following result guarantees unique solvability of the model (20).

**Theorem 1.** *For* 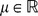 *and* 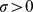 *let* 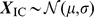 *. Then the system (20) has a unique* 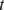 *-continuous solution* 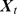 *.*

*Proof*. If we show boundedness and Lipschitz continuity of 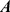 and 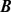 on 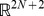, then existence and uniqueness of a solution to (20) follows from Theorem 5.2.1 in [22].

We start by proving that 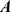 and 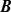 are Lipschitz continuous. Obviously 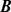 as a constant trivially satisfies a Lipschitz condition. Since linear and trigonometric functions are differentiable (and thus Lipschitz continuous), we only have to show that the Michaelis-Menten kinetics equation (12) is Lipschitz continuous with respect to dopamine. Thus, for 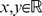 a straight-forward calculation yields 
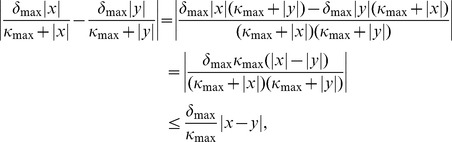
(28)where we used the reverse triangle inequality and the fact that 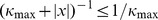 for all 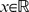. Hence, 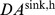 is Lipschitz continuous in 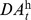 and thus all component functions of 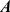 are Lipschitz which makes the entire mapping 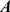 Lipschitz continuous on 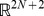.

Next, we show that 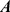 and 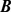 satisfy 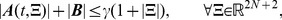
(29)where 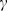 is a positive constant and 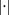 denotes some vector norm on 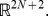 Since all norms on a finite dimensional linear space are equivalent, we prove (29) for the maximum norm 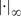. We start by showing boundedness of all components of 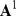 given by the right hand side of (9) with LMC equations (18). First, note that all firing rates (4) are bounded by 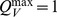 and 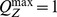 respectively. Furthermore, the rates of open ion channels defined by (3) and (14) respectively are bounded by 1. Thus, the neural activation function for potassium channels (14) satisfies 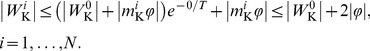
(30)


Weights connected to region 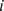 may be estimated by 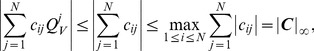
(31)where 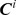 denotes the *i*-th row of the matrix 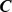. Thus, let 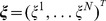 be a vector in 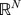 and consider the *i*-th component 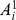 of 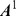 as given by the right hand side of (9) with LMC components (18). To simplify notation we introduce a vector 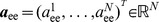 such that 
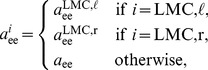
(32)with 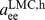 given by (16) for 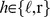. Hence, by (17), all components 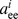 of 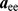 satisfy 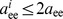. Thus we obtain the following estimate for the first term of 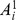


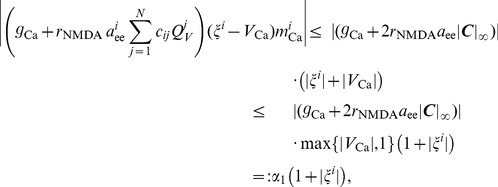
(33)where we used (31) and the fact that 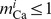. Note that all terms subsumed in the constant 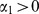 are independent of 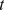 and 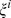. Similarly, we establish 
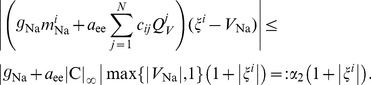
(34)


Using (30) we further obtain 
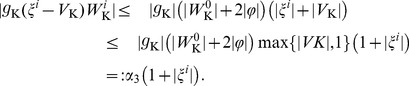
(35)


Finally, we establish 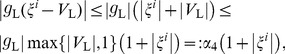
(36)and due to 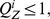


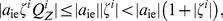
(37)for 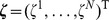. Thus combining (33) - (37) yields 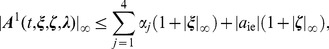
(38)where 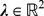. Analogously to (37) we compute 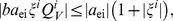
(39)and hence readily obtain 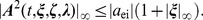
(40)


Finally, by (12) we have 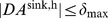, and thus we get the following estimate for (13) for any 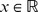


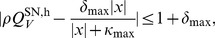
(41)where we used 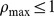 and 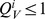. Thus we obtain 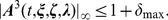
(42)


Combining estimates (38), (40) and (42) for 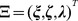 hence yields 
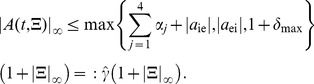
(43)


This together with the definition (7) of 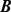 eventually gives 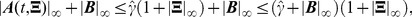
(44)which establishes (29) with 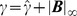 and concludes the proof. 

Having established existence and uniqueness of solutions to the model (20), we now present simulations corresponding to the resting state and dopamine modulation and compare them to empirical fMRI data.

### Simulated Temporal Brain Dynamics

Using the coupling matrix described above, brain activity was simulated corresponding to the resting state and task-induced dopamine release. A list of all used parameters is provided in Table 1, which were taken from literature and scaled appropriately to reflect units used in this work or manually estimated based on previously published values [21], [17], [31]. Physiological variations across simulated brain regions were modeled by normally distributing inhibitory-to-excitatory and non-specific-to-excitatory synaptic coupling strengths using a fixed random number generator seed across simulations. This introduced the possibility of regionally desynchronized temporal dynamics in the model allowing simulated neural nodes to evolve non-identically over time in the absence of inter-regional coupling. Note that all simulations below were run with the same initial conditions and parameter values, i.e., starting values and parameters were identical for the resting state and dopamine-modulated speech-related simulations.

**Table 1:** All parameters used in the model (including the neural and dopamine components) are provided according to their notation used in the paper, with their description, their value, and their basic units.

*Parameter*	*Description*	*Value*	*Units*
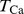	Mean opening threshold of Ca channels	−0.01	mV
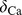	Variance of of number of open Ca channels	0.15	mV
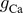	Average conductance of Ca channels	1.1	mS/ms
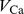	Nernst potential of Ca channels	1.0	mV
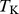	Mean opening threshold of K channels	0.0	mV
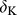	Variance of number of open K channels	0.3	mV
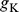	Average conductance of K channels	2.0	mS/ms
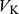	Nernst potential of K channels	−0.7	mV
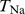	Mean opening threshold of open Na channels	0.3	mV
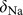	Variance of number of open Na channels	0.15	mV
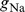	Average conductance of Na channels	6.7	mS/ms
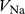	Nernst potential of Na channels	0.53	mV
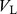	Nernst potential of leak channels	−0.5	mV
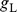	Average conductance of leaky ions	0.5	mS/ms
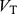	Mean potential of firing excitatory neurons	0.54	mV
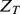	Mean potential of firing inhibitory neurons	0.0	mV
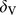	Dispersion of potential of firing excitatory neurons	2.0	mV
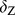	Dispersion of potential of firing inhibitory neurons	0.7	mV
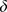	Noise current amplitude	0.3	ms^−1^
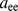	Excitatory-to-excitatory strength	0.4	mS
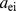	Excitatory-to-inhibitory strength	2.0	mS
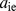	Inhibitory-to-excitatory strength	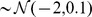	mS
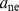	Excitatory noise input strength	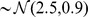	mS
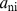	Inhibitory noise input strength	0.4	mS
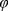	Temperature scaling factor of K channels	0.7	
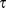	Relaxation time of K channels	1.0	ms
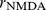	NMDA-to-AMPA strength	0.25	
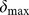	Maximum rate of dopamine re-uptake	0.004	mM/ms
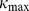	Michaelis–Menten constant	0.125	mM
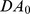	Tonic dopamine level	0.05	mM
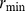	Minimum dopamine production rate	0.0005	mM/neural firing
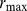	Maximum dopamine production rate	0.01	mM/neural firing
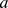	Dopamine antagonist strength	0.2	mM^−1^
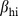	Maximum dopamine gain	50	
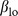	Minimum dopamine gain	1.0	
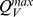	Maximum firing rate of exitatory neurons	1	kHz
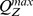	Maximum firing rate of inhibitory neurons	1	kHz
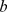	Inhibitory noise current scaling factor	0.1	

*Abbreviations*: mV  =  Millivolt, mS  =  Millisiemens, ms  =  Millisecond, mM  =  Millimole, kHz  =  Kilohertz.

In both resting-state and task simulations, complex spatio-temporal patterns of activity emerged. Fig. 1B illustrates the temporal dynamics of the left LMC with and without dopamine modulation. The left panel shows the time-course of the left LMC's excitatory membrane potential overlayed with the corresponding time-evolution of 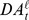. While 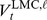 shows similar behavior during rest and task simulations in the absence of dopamine, the time-course is being visibly altered as soon as 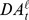 release increases. Thus, increasing dopamine levels in the task simulation changed LMC membrane potentials noticeably, which in turn raised the firing rates of LMC neurons. This increase in 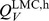 (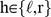) was distributed throughout the entire network, subsequently changing local neural dynamics of other brain areas. The right panel of Fig. 1B shows the time-course of 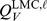 for fifty simulated speech cycles. Note that the propagation of firing rate changes acted as a neural feedback loop on the SNc itself in that repeated dopamine release caused different activity patterns than preceding cycles. In the task simulation, the LMC exhibited on average slightly higher firing rates than during rest (rest: 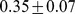, task: 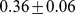, compare also Fig. 1B) in agreement with the initial modeling assumption. To highlight that the proposed dopamine release model indeed shaped the dynamics of the entire neural population, the following section discusses changes in the correlative structure of simulated brain activity under dopamine modulation relative to the resting state.

### A Simulated Functional Connectome

**Figure 2:**
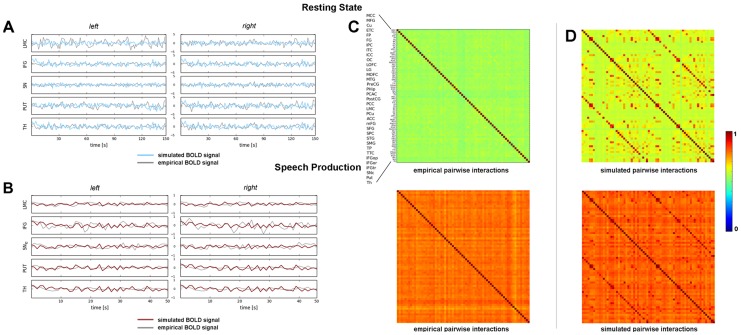
Simulated and empirical BOLD signal during (A) rest and (B) speech and NMI matrices of (C) data and (D) model in resting state and during speech production. The colored lines show time courses of simulated BOLD signals during resting state (A) and for dopamine modulation (B) for regions of the brain associated with speech production. Experimental BOLD time courses are shown in gray. The labels ‘left’ and ‘right’ indicate left and right hemispheres respectively. Pairwise interactions within the signals were quantified by computing NMI coefficients for each pair of regional time-series corresponding to the simulated and real BOLD time-courses. This gave rise to four 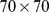 NMI-matrices (pairwise interactions of data (C) and model (D) in the resting state and during speech production). Because a normalized variant of the mutual information was employed, all matrix entries were bounded by zero and one. The parcellated brain regions used for the construction of matrices are provided in top (C) for both left and right hemispheres; the magnified inset shows the brain regions per hemisphere. *Abbreviations*: ACC/ICC/MCC/PCC  =  anterior/isthmus/middle/posterior cingulate cortex, Cu/PCu  =  cuneus/precuneus, ETC  =  entorhinal cortex, FG  =  fusiform gyrus, FP  =  frontal pole, IFGop/IFGor/IFGtr  =  pars opercularis/pars orbitalis/pars triangularis of the inferior frontal gyrus, IPC/SPC  =  inferior/superior parietal cortex, ITC/STC  =  inferior/superior temporacl cortex, LG  =  lingual gyrus, LMC  =  laryngeal motor cortex, LOFC/MOFC  =  lateral/medial orbitofrontal cortex, MFG  =  middle frontal gyrus, mFG  =  medial frontal gyrus, MTG  =  middle temporal gyrus, OC  =  occipital cortex, PCAC  =  pericalcerine cortex, PHip  =  parahippocampal cortex, PreCG/PostCG  =  pre/postcentral gyrus, Put  =  putamen, SFG  =  superior frontal gyrus, SMG  =  supramarginal gyrus, SNc  =  substantia nigra pars compacta, TP  =  temporal pole, TTC  =  transverse temporal cortex, Th  =  thalamus.



The raw model output was converted to BOLD signals as detailed above. Fig. 2 shows simulated and real BOLD signals for a selection of speech-related ROIs (Fig. 2A,B). Simulated BOLD signals with and without dopamine modulation were compared to empirical resting-state and speech production fMRI data, respectively, in order to assess the global effects of dopamine modulation on the entire simulated neural population. To do so, we employed graph theory analysis to quantify variations in functional connectivity between the resting state and speech production. Thus, we first had to quantify statistical similarity between two time-series. We chose the normalized mutual information (NMI) [47] as statistical metric. Hence, for two random variables 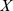 and 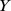, let 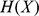 and 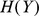 denote their respective Shannon entropies [48] and define

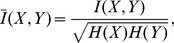
(45)where 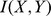 denotes the raw mutual information between 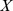 and 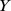. Hence, unlike the original formulation of the mutual information 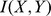, which is not bounded from above [[49], Chap. 2], the NMI is normalized by the geometric mean of the entropies 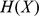 and 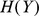. Thus, 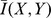 takes values between zero (two signals are independent) and one (two signals mutually depend on each other), permitting unambiguous comparison of values across data sets.

Pairwise interactions in the simulated BOLD signals with and without dopamine modulation were quantified by computing NMI coefficients for each pair of ROI time-series. Analogously, NMI matrices were computed for the group-averaged resting-state and speech production BOLD data. This gave rise to four 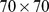 NMI-matrices (model rest, model speech, data rest, data speech) (Fig. 2C,D). Visual inspection of the matrices revealed larger variability in the model's correlative structure than in the corresponding empirical data. This might be partly explained by the fact that the empirical data were averaged across twenty subjects in an attempt to minimize subject-specific effects. Averaging a number of simulation runs would possibly decrease variability in the model; however, the aim of this study was to establish a qualitative assessment of the presented dopamine release model with respect to global effects seen in empirical data. In that respect, the proposed model, incorporating a single dopaminergic link between the SNc and laryngeal motor cortex, modulated neural activity of the whole brain to an extent that differences were observed between the structure of model's functional connectivity during dopamine release and the resting state. In addition, the model's prediction of empirical functional connectivity during speech production was in good alignment with the data.

In the following, we discuss simulated and empirical functional connectivity using the framework of graph theory. Interpreting functional connectivity matrices as graphs allowed us to not only reveal the functional topology and connectivity architecture of data and model but to also rigorously quantify the observed differences using well-established network metrics (see Supporting Information). By interpreting the 70 ROIs as nodes 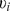 of a network with the associated NMI-coefficients representing the weights of the graph's edges, we constructed four weighted undirected graphs. Note that with the NMI being always non-negative (contrary to the classical zero-lag Pearson correlation coefficient) a graph-theoretical analysis of NMI networks is straight-forward. Without the need to either extend classical metrics to negatively weighted graphs or consider negative and positive edges separately, most graph measures can be readily applied to NMI networks.

### Graph Theoretical Analysis

The four weighted, undirected networks were analyzed following the concepts of functional integration, segregation, and influence [50]. As a measure of integration, we considered the local efficiency 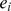 of a node 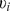, 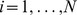, quantifying a node's local communication performance in terms of inverse shortest path lengths within its neighborhood [51]. The degree of functional segregation was estimated using the weighted local clustering coefficient 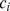, which was calculated as the average geometric mean of edge weights in triangular motifs around 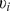 [52]. Nodal influence was approximated based on nodal strength 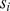 and nodal degree 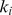. A node's strength is the sum of attached edge weights, while its degree is defined as the number of connected edges. Clustering coefficient and efficiency were also compared to corresponding values of 100 conservatively-configured, null-model random networks. Normalized clustering coefficient 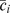 and efficiency 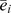 were computed by dividing 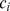 and 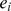 by the respective random network values. Statistical significance of differences in network metrics between the resting state and task production was determined using a paired two-sample permutation test at 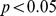 adjusted for family-wise errors (FWE) using the maximal statistic 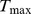 [53]. All graph metrics were calculated based on the full networks in their original density without applying any thresholding strategy. Since density-reduction techniques may severely deter network topology [54], [55], [56], [57] and might thus dilute subtle differences between simulated and empirical functional connectivity patterns, the presented analysis is focused on the full un-thresholded NMI networks as suggested by [58]. Graph metrics were computed using a Python port (pypi.python.org/pypi/bctpy) of the Brain Connectivity Toolbox for MATLAB [59].

#### Nodal influence

**Figure 3:**
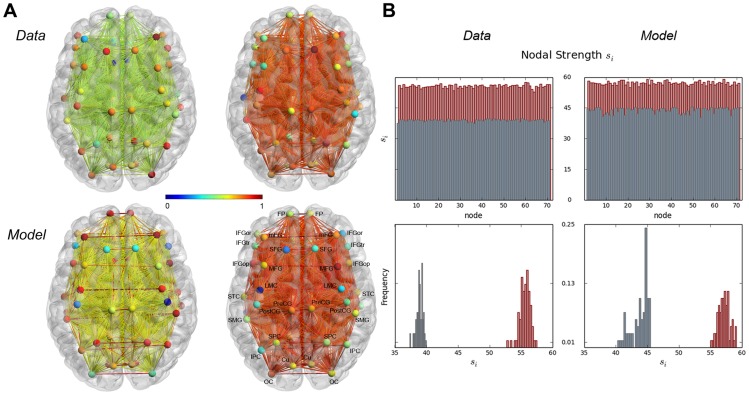
(A) Empirical and simulated functional networks in the resting state and during speech production and (B) nodal strength for experimental (left column) and simulated (right column) functional networks in resting state (gray) and during speech production (red). (A) 3D visualizations of data- and model-based NMI networks (top and bottom rows, respectively) during rest (left column) and speech production (right column). Edge colors represent NMI coefficient values and nodal color illustrates strength (normalized to the interval 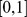). (B) Nodal strength of data- and model-based NMI networks. The top row shows the nodal strength per node, the bottom row illustrates the distribution of *s_i_*-values. The 3D networks were visualized with the BrainNet Viewer (http://www.nitrc.org/projects/bnv/). *Abbreviations*: MFG  =  middle frontal gyrus, Cu  =  cuneus, FP  =  frontal pole, FG  =  fusiform gyrus, IPC/SPC  =  inferior/superior parietal cortex, LMC  =  laryngeal motor cortex, OC  =  occipital cortex, PreCG  =  precentral gyrus, IFGop/IFGor/IFGtr  =  pars opercularis/pars orbitalis/pars triangularis of the inferior frontal gyrus, PostCG  =  postcentral gyrus, STC  =  superior temporal cortex, mFG  =  medial frontal gyrus, SFG  =  superior frontal gyrus, SMG  =  supramarginal gyrus.

Figure 3 shows nodal strengths of the networks. We found a significant increase in strength when comparing resting state to task production in both data and model (both 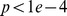). While the simulated networks showed a higher average strength than the data in the resting state (model: 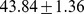; data: 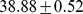), the difference was less pronounced during task production (model: 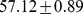, data: 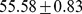). Examining the distribution of nodal strengths in the data, we observed a marked right-shift of the distribution during speech as compared to the resting state, clearly reflecting overall elevated strength in the speech production network. This right-shift was seen in the simulated networks too, although to a lesser extent. The data showed a narrower strength distribution than the model in the resting state, reflecting higher variability of NMI coefficients for the simulated BOLD signal without dopamine modulation (compare also the corresponding NMI matrices shown in Fig. 2C,D). Nodal degrees of the networks did not reveal any particular structure. All networks (model and data) were maximally connected, i.e., all nodes had maximum degree 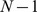, which means all pairwise NMI coefficients were non-zero. Note that, unlike Pearson's correlation coefficient (PCC), the mutual information does not only reflect linear correlation, but also dependencies in higher moments [60]. While a zero PCC only indicates that there is no linear relationship between the observed quantities, two time-series have to be approximately statistically independent for the NMI to be zero (compare, e.g., [61]). In other words, two signals have to show a stronger kind of independence to yield an NMI coefficient of zero. Given that fMRI-based functional networks are largely composed of high-strength nodes, are fully-connected, and may be indistinguishable from random networks if unthresholded [62], it was not surprising to see overall positive NMI coefficients for the data. It was also expected that simulated BOLD signals generated by a system of coupled but structurally identical equations show large NMI coefficients.

#### Network segregation

**Figure 4:**
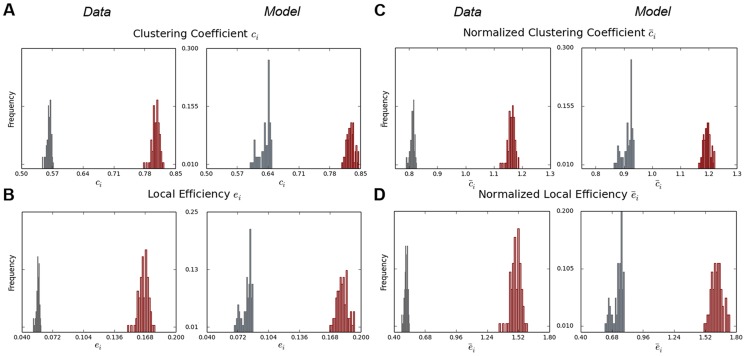
Non-normalized and normalized segregation and integration metrics for experimental and simulated functional networks in resting state (gray) and during speech production (red). Distributions of (A) non-normalized clustering coefficient, (B) non-normalized local efficiency, (C) normalized clustering coefficient, and (D) normalized local efficiency in the data- and model-based NMI networks.

As mentioned above, the local clustering coefficient 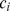 quantifies the average weight of connected neighbors of the node 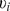. The networks considered here had maximal connection density, i.e., each node was connected to all other nodes in the graph. In this case, 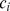 is not influenced by the presence or absence of edges and is thus given by the geometric mean of 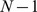 edge weights adjacent to 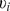. Hence, the local clustering coefficient is solely dependent on the nodal strength. Thus, 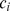 (Fig. 4A) exhibited qualitatively the same characteristics as 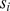 (compare to Fig. 3B). In both data and model, we observed a significant increase in clustering during task production as compared to rest (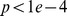) (data: rest: 0.56
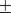
0.01, speech: 0.81
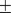
0.01; model: rest: 0.63
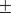
0.01, speech: 0.83
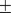
0.01) Interestingly, compared to the data, the model showed on average higher values of 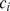 in the resting-state simulation, while the dopamine-modulated run exhibited very similar clustering characteristics. To assess differences in network topologies in contrast to random graphs, we compared 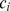 to the corresponding random network values and computed the normalized clustering coefficient 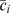 (Fig. 4C). We found 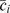 to be greater than one in the dopamine modulated simulation and the empirical speech production networks, while both data and model failed to show values larger than one during rest (data: rest: 0.81
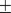
0.01, speech: 1.16
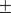
0.01; model: rest: 0.91
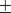
0.02, speech: 1.19
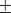
0.01). This indicated an overall elevated segregation of simulated as well as empirical speech production networks in relation to random networks. Furthermore, for both simulated and empirical networks the difference in values of 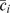 between rest and task was found to be significant (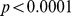). Interestingly, with and without dopamine modulation the model showed a very similar variability in both 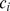 and 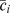 compared to the empirical networks. However, while the data exhibited similar peak frequencies during rest and speech, a decrease in the most prevalent values of both 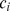 and 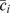 was found in the simulated networks (Fig. 4A,C). This was indicative of a slightly lower variability of 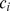 and 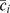 in the dopamine modulated simulation.

#### Network integration

We considered functional integration of the NMI networks by evaluating values of local efficiency 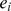 (Fig. 4B). For fully connected networks, 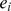, similar to the clustering coefficient, is completely determined by the nodal strength, since the shortest path between two nodes is always given by their connecting edge. Thus, 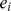 showed similar characteristics as 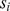. We saw a statistically significant increase during task production as compared to the resting state (all 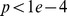) with the model showing on average higher values of 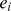 (data: rest: 0.06
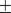
0.001, speech: 0.17
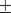
0.005; model: rest: 0.08
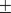
0.005, speech: 0.18
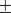
0.006). We normalized 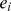 to analyze differences in network integration compared to a set of comparable random graphs. We found significant differences in local efficiency between both data and model during rest and speech (all 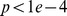). However, similar to the clustering coefficient, simulated as well as empirical networks showed on average a normalized efficiency smaller than one during the resting state, while data and model exhibited values larger than one during task production (data: rest: 0.51
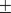
0.01, speech: 1.50
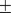
0.04; model: rest: 0.73
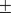
0.05, speech: 1.62
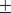
0.05). Simulated and empirical networks showed a significant (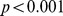) increase in normalized efficiency when transitioning from rest to task, which was indicative of larger nodal integration in the networks.

## Discussion

We presented an extension of a model of neural assemblies proposed by Breakspear et al. [21] to simulate dopamine release in the human brain during complex voluntary behaviors. In contrast to other large-scale neural modeling techniques based on coupled oscillator systems, our approach was grounded in neuroanatomy and physiology and thus allowed us to design a dopamine release model guided by biological considerations. We established unique solvability of the proposed model and demonstrated a computationally efficient strategy to numerically approximate its solutions.

In the context of the model, we assumed the difference between the resting state and speech production to be solely given by a modulation of dopamine levels in the LMC via a direct input from the SNc. Thus, the model was oblivious to task-related effects caused by any neurotransmitter other than dopamine. Importantly, we observed pronounced differences between the resting state and task production in simulations. This finding may be interpreted as an indication of the profound physiological impact of dopamine on brain dynamics.

It is remarkable that altered neural firing rates within the bilateral LMC only were sufficient for the entire simulated neural population to exhibit changes in its temporal dynamics. We attribute these observed task differences to dopamine driving neural dynamics via the coupling matrix 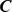. Given real structural connectivity data as input, the strength of the model lies in its ability to reproduce observed properties of connectivity during a dopamine-modulated activity with a biophysical prescription for dopamine neurotransmission.

To quantify the impact of dopamine release on the entire neural mass, we used functional connectivity and graph theory analysis and interpreted the results as a stationary synopsis of the global effects of dopamine modulation. The graph theoretical analysis of the functional connectomes revealed a number of similarities between the model and data. Due to the slightly higher variability of NMI coefficients, especially during the rest, (Fig. 2) nodal strengths of the model without dopamine modulation showed more fluctuations than corresponding values for the data. Nonetheless, strength values of simulated and empirical networks were in good agreement with the model exhibiting slightly larger values. Clustering coefficients of both model and data also showed qualitatively similar attributes, when comparing the resting state to dopamine modulation. Thus, the model showed characteristics comparable to those of the data with respect to functional segregation and nodal influence.

Similarly, local efficiency showed good qualitative agreement between simulated and empirical connectomes, thus the model mimicked functional integration patterns seen in the experimental data. However, both model and data failed to show increased network segregation and integration compared to random graphs during rest but showed consistently larger values than null model networks during speech production (all 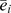 and 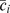 greater than one for 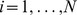). This may support earlier findings indicative of pronounced changes in network organization for speech control [63], [64]. It should be emphasized that normalized efficiency is computed using the notion of shortest paths within a network. Due to the absence of zero-weighted edges in the considered networks, the shortest path between any two nodes in the graphs was given by their connecting edge, effectively side-stepping the notion of paths in a graph. A thresholding strategy to eliminate ‘weak’ edges (i.e., edges corresponding to small NMI coefficients) may have somewhat remedied this problem. It should be noted, however, that interpreting efficiency values obtained from functional networks that are based on statistical similarity between brain areas is not an immediately evident approach. Indeed, since NMI networks express not only direct but also all indirect couplings between regions, a path-based metric, like efficiency, may yield ambiguous results (see, e.g., [65], [66]).

Nevertheless, decreasing connection densities in the networks would also yield non-trivial nodal degree distributions, opening another perspective on nodal influence within the networks. However, weight-based thresholding must be performed with considerable precautions so as to not deteriorate topological properties of a network. Since this work was mainly concerned with establishing a biologically-informed, large-scale model with optional dopamine neuromodulation, no thresholding strategy was applied to the constructed functional networks. A future study focused exclusively on the graph-theoretical analysis of functional networks should address this issue.

### Limitations

A visual inspection of the simulated and empirical functional connectomes (Fig. 2) revealed that the model tended to slightly overestimate regional pairwise interaction during both resting state and dopamine modulation. This finding was not surprising considering the fact that the simulated BOLD signals were generated by 140 structurally identical equations that only differed in some parameter values. This is an apparent limitation of the presented approach. However, one of the advantages of the presented model is that the employed strategy enabled us to perform large-scale simulations of brain activity based on considerable neurobiological detail without becoming too complex to be practically unfeasible.

Coupling between regions in the model was achieved via scaling excitatory neural firing rates by entries of the coupling matrix 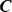 (compare eqn. (9)). Thus, all modeled axonal connections were excitatory, which is a simplification that ignores the effects of feedforward inhibition. In particular, firing of connected areas impacted a region's membrane potential through excitatory projections targeting local populations of NMDA and AMPA receptors. In other words, inter-regional coupling was not modeled as an explicit consequence of changes in neural voltages of neighboring areas. Instead, the influence of other regions on the local membrane potential was mediated by changes in neural firing rates. In this context it should be noted that the proposed model did not include an explicit representation of inter-regional axonal conduction delays. To some extent, however, the employed form of indirect coupling in the model may be interpreted as a lumped representation of conduction time delays.

### Future Directions

Having tested the model for its efficacy in reproducing essential features of real data from healthy humans during speech production, the next step should be an examination of clinical relevance of the proposed neural population model. This may be achieved by incorporating ‘lesions’ into a simulated network of interest to investigate the extent of inter-regional influences coupled with dopaminergic transmission in a range of neurological and psychiatric disorders, such as Parkinson's disease, dystonia, schizophrenia, etc.

Furthermore, the model's use is not limited to human applications [67] and may be applied equally well to animal models of disease and normal behavior, taking into account appropriate modifications for differences in animal and human dopaminergic innervation [68].

Since most parameters used here were taken from literature, some inferences about trajectories of isolated regions can be formulated based on the exhaustive analytical treatment of the original model [21]. In the original model, inter-regional coupling is introduced using a scalar parameter 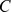 that acts on the spatially averaged excitatory firing rates of all modeled nodes, i.e., 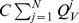. The approach presented here uses not a scalar, 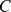, but a matrix, 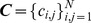, to introduce coupling and thus expands the scaled mean field firing to be 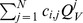. This can be seen as a weighted average of firing rates. Thus, in the absence of dopamine and for a diagonal coupling matrix 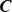 the dynamic behavior of an isolated node can be reduced to the cases discussed by Breakspear et al. [21]. However, for a general non-diagonal coupling matrix, the dynamics become increasingly more complex. Moreover, as our simulation results indicated, dopamine modulation also had a pronounced impact on the overall behavior of the model. Thus, the extensions proposed here changed the dynamics of the original model in a non-trivial way. Thus, a rigorous dynamical analysis of the presented model would require a thorough study of the non-linear relation between 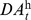 and 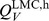 (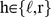) and an assessment of the influence of dopamine-related parameter choices on the temporal evolution of the LMC nodes in terms of a full sensitivity analysis [69], [70], [71]. It was not within the scope of this work to present such an exhaustive analytical treatment of the model. Nonetheless, this poses an interesting direction for future studies.

Given the demonstrated differences in functional connectivity across the entire experimental time in simulations of resting versus speech conditions, the question arises as to what extent dopamine altered function on small versus long time scales within the tasks. Our results indicate that dopamine may influence dynamics on long time scales. This may suggest that rapid temporal release of dopamine, as evidenced by the spontaneous dopamine release incorporated during each time-step in the model, may be involved in slow plastic responses. Thus, it is tempting to speculate that a future adaption of the proposed dopamine model might yield further insight into the learning and adaptation involved in voluntary behaviors, particularly given dopamine's involvement in learning and motivational behavior in other tasks.

Finally, models simpler than the one considered in this paper are capable of reproducing empirical functional connectivity. In fact, a recent study showed that a stationary model of resting-sate functional connectivity explains functional connectivity better than more complex models [72]. In modeling empirical functional connectivity as accurately as possible, the application of a complexity reduction technique [73] to the introduced highly non-linear model should be considered in order to derive a set of considerably simpler equations of statistical moments. On the other hand, it has been shown [73] that functional connectivity is essentially state-dependent and that local changes of activity in a set of cortical areas (due to external inputs, attention, neuromodulation, or learning) change the dynamical state of the brain network, thus modifying the correlations between the brain areas and introducing various levels of complexity. Along this line, while simpler models have a number of computational advantages (e.g., reduced computational load, easier estimation of parameters, simpler relationship between structure and function), their ability to simulate complex temporal activity patterns at various cognitive scales (and in the context of simulated dopamine modulation) may be somewhat limited. This motivated the development of the proposed complex model to better understand empirical data and to make predictions about the different states of dopamine-modulated brain activity during voluntary behavior. Future work should be directed to a possible simplification of this model, while assuring its ability to accurately reproduce the complex biological patterns of voluntary behaviors.

### Summary

We conclude that a regional model that includes dopamine release, reuptake, and modulation of ion channels significantly alters the behavior of an otherwise unmodulated, resting state neural population model. This work thus combines a small-scale basic cellular biology understanding of dopamine to alter macroscopic behavior of neuronal systems with nontrivial structural circuity, and presents meaningful global simulated fMRI network behavior. Region-specific analysis warrants the identification of specific effects of neuromodulation on task-based networks for speech and other dopamine-modulated voluntary behaviors.

## Supporting Information

Text S1 **Hemodynamic model and network metrics.** Details regarding the hemodynamic model used to convert the raw model output to BOLD signals as well as a short description of the used network metrics and the construction of the random networks.
(PDF)
