# Supplementary material for: A Neural Population Model Incorporating Dopaminergic Neurotransmission during Complex Voluntary Behaviors
Source: PLoS Comput Biol. 2014 Nov 13;10(11):e1003924. doi: 10.1371/journal.pcbi.1003924 (PMC4230744; doi:10.1371/journal.pcbi.1003924)
Supplement: Text S1 — Hemodynamic model and network metrics. Details regarding the hemodynamic model used to convert the raw model output to BOLD signals as well as a short description of the used network metrics and the construction of the random networks. (PDF) [file pcbi.1003924.s001.pdf]

Supporting Information

Hemodynamic Response Model

To reduce computational load, the raw regional neural voltages with a temporal resolution of 0.1ms generated by the model were down-sampled to 100Hz. We also observed that higher temporal resolutions did not alter the dynamics of the simulated BOLD time-series. In order to be able to meaningfully compare the simulated signals to the considered fMRI data, the model was run for the same period of time the data was recorded in. For instance, the resting state data was acquired over a period of five minutes. Thus, 3 000 000 ms were simulated to obtain a comparable signal. Following [1] a linear hemodynamic response function was employed:

$$\psi(t) = \Psi_1 e^{-t/\vartheta_1} t^{\alpha_1 - 1} - \Psi_2 e^{-t/\vartheta_2} t^{\alpha_2 - 1}, \quad (1)$$

where we used $\Psi_1 = 0.02, \Psi_2 = 2.34e - 8$ as amplitudes of the filter, $\vartheta_{1,2} = 0.9$ as temporal delays, and the scaling factors $\alpha_1 = 7.98, \alpha_2 = 13.97$. Regional neural voltages V_t^i were thus transformed to BOLD signals using the convolution $\psi * V^i$. Note that the amplitudes Ψ_i of the BOLD filter ψ were not fit to the data to avoid any undue influence on the auto-correlative structure of the model. However, if the scope of a study were to accurately reproduce a given BOLD signal, such a fitting procedure should be employed to maximize alignment between model and data. Finally, the convoluted signal was sub-sampled to match recording parameters of the data.

Null-Model Random Network Construction

The values of most network measures strongly depend on a graph's size (i.e., the number of nodes), its density and its degree distribution [2, Chap. 2]. Therefore, the significance of differences in metrics is usually assessed by comparing values to ones calculated for null-hypothesis networks, which are based on the original graphs but constructed to exhibit random topologies [3]. In this work, the random graphs were based on the NMI matrices shown in Fig. 2 and constructed to preserve the degree-, weight- and strength- distributions of the original networks.

Graph Metrics

In the following, we give a brief description of the network metrics used in this paper. A comprehensive review of graph measures and their applications for the understanding of normal and diseased brain states is given in [2, 3].

Every graph G consists of a set of nodes (vertices) and edges (links), formally written as $G = \langle V, E \rangle$. In case of the functional brain networks discussed here, the nodes are brain areas, i.e., points in three-dimensional space. Thus, we introduce the set of nodes $\boldsymbol{V} = \left\{ \boldsymbol{v}_i \in \mathbb{R}^3 \,|i = 1, \ldots, N \right\}$, where N denotes the number of considered regions. Because functional connectivity networks are undirected, we only consider edges without orientation, i.e., the edge connecting nodes $\boldsymbol{v}_i$ and $\boldsymbol{v}_j$ is the same as the link from $\boldsymbol{v}_i$ to $\boldsymbol{v}_j$ (incoming and outgoing connections are identical). Hence, we write the edge connecting $\boldsymbol{v}_i$ and $\boldsymbol{v}_j$ as unordered pair $\{\boldsymbol{v}_i, \boldsymbol{v}_j\}$ or $\boldsymbol{v}_i \leftrightarrow \boldsymbol{v}_j$ and thus we define the set of all edges in the undirected graph as $\boldsymbol{E} = \{\boldsymbol{v}_i \leftrightarrow \boldsymbol{v}_j \,|1 \leq i, j \leq N\}$. Hence, we consider the graph $\boldsymbol{G} = \langle \boldsymbol{V}, \boldsymbol{E} \rangle$. Let further $\{a_{ij}\}_{i,j=1}^N =: \boldsymbol{A} \in \mathbb{R}^{N \times N}$ denote the graph's adjacency matrix such that

$$a_{ij} = \begin{cases} 1, & \text{if } \boldsymbol{v}_i \leftrightarrow \boldsymbol{v}_j \in \boldsymbol{E}, \\ 0, & \text{otherwise.} \end{cases} \tag{2}$$

The considered networks are weighted undirected networks, thus each edge $\boldsymbol{v}_i \leftrightarrow \boldsymbol{v}_j$ is associated with a weight $w_{ij} = w_{ji}$. Hence we introduce a mapping $W : \boldsymbol{E} \to \mathbb{R}$ given by

$$W\left(\boldsymbol{v}_i \leftrightarrow \boldsymbol{v}_j\right) = w_{i,j}, \quad 1 \leq i, j \leq N, \tag{3}$$

and collect all edge weights in a (real symmetric) $N \times N$ matrix $\boldsymbol{W} = \{w_{ij}\}_{i,j=1}^N$ (where we set $w_{k\ell} = 0$ if there exists no edge between $\boldsymbol{v}_k$ and $\boldsymbol{v}_\ell$). Based on these quantities a number of network metrics can be computed.

Nodal Influence Metrics estimating nodal influence try to quantify a single node's importance in the network. One of the simplest influence measures is the *degree* of the node $\boldsymbol{v}_i$ given by (compare, e.g., [2, Chap. 2])

$$k_i = \sum_{j=1}^N a_{ij}, \quad i = 1, \ldots, N,$$

where a_{ij} denote the entries of the adjacency matrix $\boldsymbol{A}$ defined in (2). The weighted version of the degree is the nodal *strength* [3]

$$s_i = \sum_{j=1}^{N} w_{ij}, \quad i = 1, \ldots, N,$$

with w_{ij} denoting edge weights.

Network Integration Integration measures are designed to estimate a network's predilection for system-wide interaction. Most of these metrics are based on the concept of paths. A path $\boldsymbol{p}_{i\leftrightarrow j}$ from node $\boldsymbol{v}_i$ to $\boldsymbol{v}_j$ in the graph $\boldsymbol{G}$ is a sequence of nodes $\boldsymbol{p}_{i\leftrightarrow j} = \{\boldsymbol{v}_i = \boldsymbol{v}_{i_1}, \boldsymbol{v}_{i_2}, \ldots, \boldsymbol{v}_{i_n} = \boldsymbol{v}_j\}$ such that $\boldsymbol{v}_{i_k} \leftrightarrow \boldsymbol{v}_{i_{k+1}} \in \boldsymbol{E}$ for $k = 1, \ldots, n$. The shortest (weighted) path $\bar{\boldsymbol{p}}_{i\leftrightarrow j}$ between $\boldsymbol{v}_i$ and $\boldsymbol{v}_j$ is the path with minimal total edge weight, also called (weighted) shortest path length

$$d_{ij} = \sum_{\{\boldsymbol{v}_{i_k}, \boldsymbol{v}_{i_{k+1}}\} \in \bar{\boldsymbol{p}}_{i\leftrightarrow j}} W\left(\boldsymbol{v}_{i_k} \leftrightarrow \boldsymbol{v}_{i_{k+1}}\right),$$

where we used the mapping W defined in (3). The *efficiency* e_i of a node $\boldsymbol{v}_i$ estimates the extent of possible interaction in a neighborhood around $\boldsymbol{v}_i$ in terms of the inverse shortest path length [4]

$$e_i = \frac{1}{N-1} \sum_{\substack{j=1 \\ j \neq i}} \frac{1}{d_{ij}}, \quad i = 1, \ldots, N.$$

Network Segregation A segregated network is organized into local communities. One of the most widely used measures to quantify segregation of a graph is the *clustering coefficient* [5]. The clustering coefficient c_i of a node $\boldsymbol{v}_i$ is based on the geometric mean of link weights in triangles around $\boldsymbol{v}_i$

$$c_i = \frac{2}{k_i(k_i-1)} \sum_{j,k=1}^{N} \left(w_{ij} w_{jk} w_{ki}\right)^{1/3}, \quad i = 1, \ldots, N,$$

where k_i denotes the nodal degree.

References

1. Glover G (1999) Deconvolution of impulse response in event-related bold fmri. NeuroImage 9: 416–429.

2. Sporns O (2011) Networks of the Brain. MIT Press.

3. Rubinov M, Sporns O (2010) Complex network measures of brain connectivity: uses and interpretations. NeuroImage 52: 1059–1069.

4. Latora V, Marchiori M (2001) Efficient behavior of small-world networks. Physical Review Letters 87.

5. Onnela JP, Saramäki J, Kertész J, Kaski K (2005) Intensity and coherence of motifs in weighted complex networks. Physiscal Review E 71.
